# Optical Fiber Tweezers: A Versatile Tool for Optical Trapping and Manipulation

**DOI:** 10.3390/mi11020114

**Published:** 2020-01-21

**Authors:** Xiaoting Zhao, Nan Zhao, Yang Shi, Hongbao Xin, Baojun Li

**Affiliations:** Institute of Nanophotonics, Jinan University, Guangzhou 511443, China; xiaotingzhao@stu2019.jnu.edu.cn (X.Z.); zhaonan@stu2018.jnu.edu.cn (N.Z.); shiyang@jnu.edu.cn (Y.S.); baojunli@jnu.edu.cn (B.L.)

**Keywords:** optical fiber tweezers, optical trapping and manipulation, optical force, photothermal effect, evanescent fields, cell trapping and assembly

## Abstract

Optical trapping is widely used in different areas, ranging from biomedical applications, to physics and material sciences. In recent years, optical fiber tweezers have attracted significant attention in the field of optical trapping due to their flexible manipulation, compact structure, and easy fabrication. As a versatile tool for optical trapping and manipulation, optical fiber tweezers can be used to trap, manipulate, arrange, and assemble tiny objects. Here, we review the optical fiber tweezers-based trapping and manipulation, including dual fiber tweezers for trapping and manipulation, single fiber tweezers for trapping and single cell analysis, optical fiber tweezers for cell assembly, structured optical fiber for enhanced trapping and manipulation, subwavelength optical fiber wire for evanescent fields-based trapping and delivery, and photothermal trapping, assembly, and manipulation.

## 1. Introduction

Optical forces have been widely used for optical trapping and manipulation of particles while using laser beams since 1970, when Arthur Ashkin used two counter-propagating and continuous wave focused beams to trap particle [1]. In 1986, Ashkin and co-workers used a single tightly focused laser beam, which realized the stable trapping of particles. They then named the optical-trapping technique as optical tweezers [2], which we refer to conventional optical tweezers (COTs). Over the years that followed, Ashkin and co-workers carried out a series of studies, which not only realized the capture of particles ranging from tens of nanometers to tens of microns with a single focused beam, but also realized the capture of viruses and bacteria [2,3].

**Figure 1:**
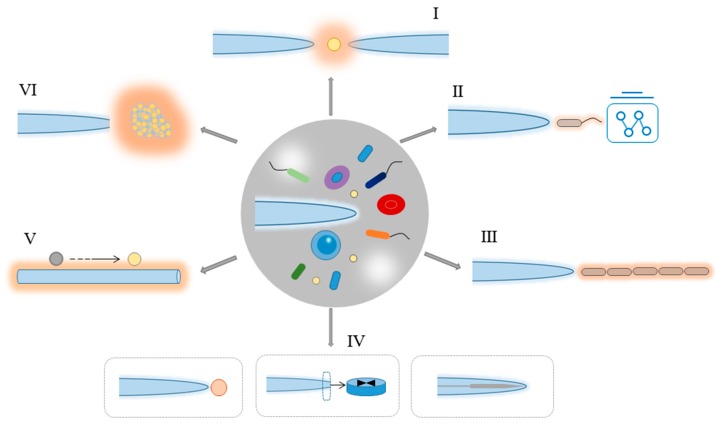
The overview figure of optical fiber tweezers (OFTs) for particle and cell trapping and manipulation. (**I**) Dual fiber tweezers for trapping and manipulation; single optical fiber tweezers for (**II**) cell trapping and single cell analysis, and (**III**) cell assembly; (**IV**) structured optical fiber tweezers for trapping and manipulation; (**V**) subwavelength optical fiber wire for evanescent fields-based trapping and delivery; and, (**VI**) optical fiber-based massive photothermal assembly and manipulation.

After nearly 50 years of development, optical trapping and manipulation via COTs have made great progresses in both methods and applications, with manipulated samples ranging from different dielectric particles to biological cells and biomolecules [4,5]. However, a high numerical aperture (NA) objective is necessary for the light focusing via COTs. In addition, different optical components for beam expanding and steering are also essential. This bulky structure makes it lack manipulation and control flexibility. Alternatively, in 1998, holographic optical tweezers (HOTs) that can realize multiple traps with complex structured light fields have been developed [6]. These multiple traps can be achieved by means of computer generated holograms via the modulation of spatial light modulators. Such HOTs greatly increase the manipulation and control degrees, and they are also widely used for multiple particle trapping and manipulation [7,8,9,10]. However, it is difficult for the stable trapping of particles in the nanometer scale due to the diffraction limit. In the late 2000s, surface plasmon-based optical tweezers (SPOTs) [11,12] have been created, which can realize the stable trapping of nanoparticles, even single molecules with a few nanometer scale, to realize the nanoscale trapping and manipulation [13,14]. Though with the ability for the trapping of nanoscale particles, the SPOTs necessitate carefully designed and elaborate nanostructures. These nanostructures also limit the manipulation flexibility. Techniques, such as COTs, HOTs, and SPOT, involve complex devices and components with inflexible control. Therefore, it is particularly important to seek a simple and flexible control and manipulation tool. The development of optical fiber tweezers (OFTs) [15,16] makes it a versatile candidate for optical trapping and the manipulation of different samples. OFTs possess exceptional advantages in the manipulation flexibility due to the simple structure with only optical fibers. The fiber can be inserted into thick samples and turbid media, which greatly increases the sample applicability. In addition, OFTs make optical manipulation a low cost technique due to the easy fabrication procedures. OFTs can also be integrated into small devices, such as optofluidic channels and chips [17]. OFTs were first presented in 1993, where two aligned single-mode optical fibers were used for optical trapping [18]. While tiny particles and cells can be directly captured and manipulated using the optical scattering force generated by two fibers [19], the manipulation flexibility is limited by the two fibers. Indeed, a single optical fiber can also be used for particle trapping and manipulation. In 1997, the first optical manipulation using a single tapered optical fiber was reported [20]. These single fiber-based OFTs greatly increase the manipulation flexibility. The end of a single fiber for light focusing is similar to that in COTs after being drawn into a lenticular shape, which creates a stronger gradient force on the particle and makes it easier for optical trapping [21,22,23]. Both dual and single fibers can be used for stable trapping, as shown in Figure 1.

OFTs can be divided into two categories, according to their working principles: photothermal effect and optical force. The former generates trapping force by laser-induced thermal and acoustic gradients in the surrounding medium of the particles [24], while the latter has been traditionally decomposed into two components: the scattering force in the direction of light propagation and the gradient force in the direction of the optical intensity gradient [25]. 

For the purpose of this review, we will focus on the OFTs and summarize their recent advances in the trapping and manipulating of tiny objects, especially for cells.

## 2. Theoretical Analysis of Optical Forces Exerted on Particles

Optical force will be exerted on the particles near the focal point by the momentum transfer from the scattering of incident photons when a high intensity laser beam is irradiated on dielectric particles. The resulting optical force traditionally consists of two components: a scattering force and a gradient force. The Rayleigh scattering condition will be satisfied when the wavelength of laser beam is much longer than the size of trapped particle [25]. With the above condition, the optical force can be obtained by treating particles as point dipoles. For a particle of radius *a*, this force is given by

(1) $$F_{\mathrm{scatt}}=\frac{I_{0}\sigma n_{m}}{c}$$

(2) $$\mathrm{With}\;\sigma =\frac{128\pi^{5}a^{6}}{3\lambda^{4}}{\left(\frac{m^{2}-1}{m^{2}+2}\right)}^{2}$$

where *I_0_* is the intensity of the incident light, *σ* is the scattering cross section of the particle, *n*_m_ is the refractive index of the surrounding medium, *c* is the speed of light in vacuum, *m* is the ratio of the refractive index of the particle to that of the medium (*n_p_*/*n*_m_), and *λ* is the wavelength of the trapping laser. The scattering force is in the same direction as the incident light and it is proportional to the intensity of the light. The optical gradient force is generated by the interaction between the induced dipole and the non-uniform field, as given by

(3) $$F_{\mathrm{grad}}=\frac{2\pi \alpha }{cn_{m}{}^{2}}\nabla I_{0}$$

(4) $$\mathrm{with}\;\alpha =n_{m}{}^{2}a^{3}\left(\frac{m^{2}-1}{m^{2}+2}\right)$$

However, for many cases, the size of the trapped particles is comparable to the wavelength of the trapping laser beam. In this situation, the point-dipole approach is invalid. More complete electromagnetic theories are necessary for the force calculation. Fortunately, numerical simulation and calculation methods that are based on electromagnetic theories, such as the finite element method and finite difference time domain method, can be used for the calculation of optical forces. The total optical force (***F***_O_) exerted on the particle can be calculated by calculating the integral of the time-independent Maxwell stress tensor (<**T**_M_>) along the total external surface of the particle. <**T**_M_> can be expressed as

(5) $$\langle T_{M}\rangle =DE^{*}+HB^{*}-1/2\left(D\cdot E^{*}+HB^{*}\right)I$$

where **D** and **H** are the electric displacement and magnetic field, respectively; E* and B* are the complex conjugates of the electric field **E** and magnetic flux field **B**, respectively; and, **I** is the isotropic tensor. **F**_O_ can be expressed as

(6) $$F_{O}=\oint_{s}\left(\langle T_{M}\rangle \cdot n\right)dS$$

where **n** is the surface normal vector.

## 3. Dual Fiber Tweezers for Trapping and Manipulation

**Figure 2:**
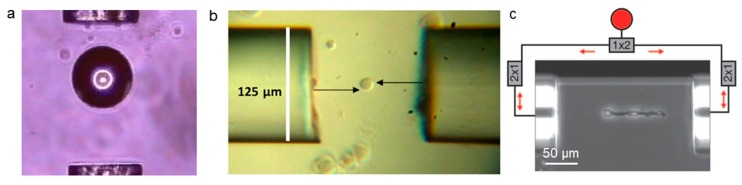
Dual fiber tweezers for cell trapping and manipulation. (**a**) A 100 µm polymer is trapped by the dual fibers. Adapted with permission from Jess et al. [27]. (**b**) The human smooth muscle cell is captured and rotated by optical fiber at the center of two horizontally biased fibers (20 mW in each arm). Adapted with permission from Black et al. [29]. (**c**) The Müller cell is captured, aligned, and stretched by two near-infrared laser beams that propagate backwards from the fiber. Adapted with permission from Franze et al. [32].

The dual fiber tweezers mainly use the scattering force to trap particles cooperatively by two optical fibers. It can form a large enveloping area and easily accommodate large objects due to its beam divergence. In addition, the dual fiber tweezers do not use focused light, resulting in minimal radiation damage to living cells [26]. Jess et al. used a fiber arm with an output power of 800 mW to trap a 100 µm polymer sphere [27] (Figure 2a). Decombe et al. achieved the capture of a 1 μm polystyrene sphere at an optical power of 2 mW by using optical tweezers with the tips of two chemically corroded fibers [28].

The rotation of the trapped particles can also be achieved by the dual fiber tweezers. For example, a fiber spanner tool [29], which was formed by two transversely offset fibers with counterpropagating laser beams that can rotate particles, like a spanner tool, was used to introduce the lateral offset between the two back-propagating divergent beams from the single-mode fiber. The center of the fiber is laterally biased and the optical fiber is used to trap and rotate the human smooth muscle cell (hSMC) (Figure 2b). If there is a zero offset, the object can remain in a static state. The object begins to rotate when two fibers have a relative displacement [29]. The rotation of living cells can be performed in a dual-beam optical fiber trap integrated into a modular laboratory chip system [30].

Alternatively, the cells can be stretched after being captured with a double beam. Guck et al. constructed a device and named it an optical stretcher [31]. The principle of the optical stretcher is as follows: when placing the dielectric object in the middle of two opposed, non-focused laser beams, the total force acting on the object is zero, but the surface forces are additive, which causes the object to be stretched along the beam axis. As shown in Figure 2c, Müller cell can be trapped, aligned, and stretched out by two counter-propagating near-infrared laser beams diverging from the optical fibers, where the incident laser wavelength is 1064 nm [32]. The cell stretching results from the additive surface force on the cell membrane due to momentum changes of photons [31]. The red blood cells can also be stretched using dual-trap optical tweezers after placing them in the suspension and expanding into a sphere [33].

**Figure 3:**
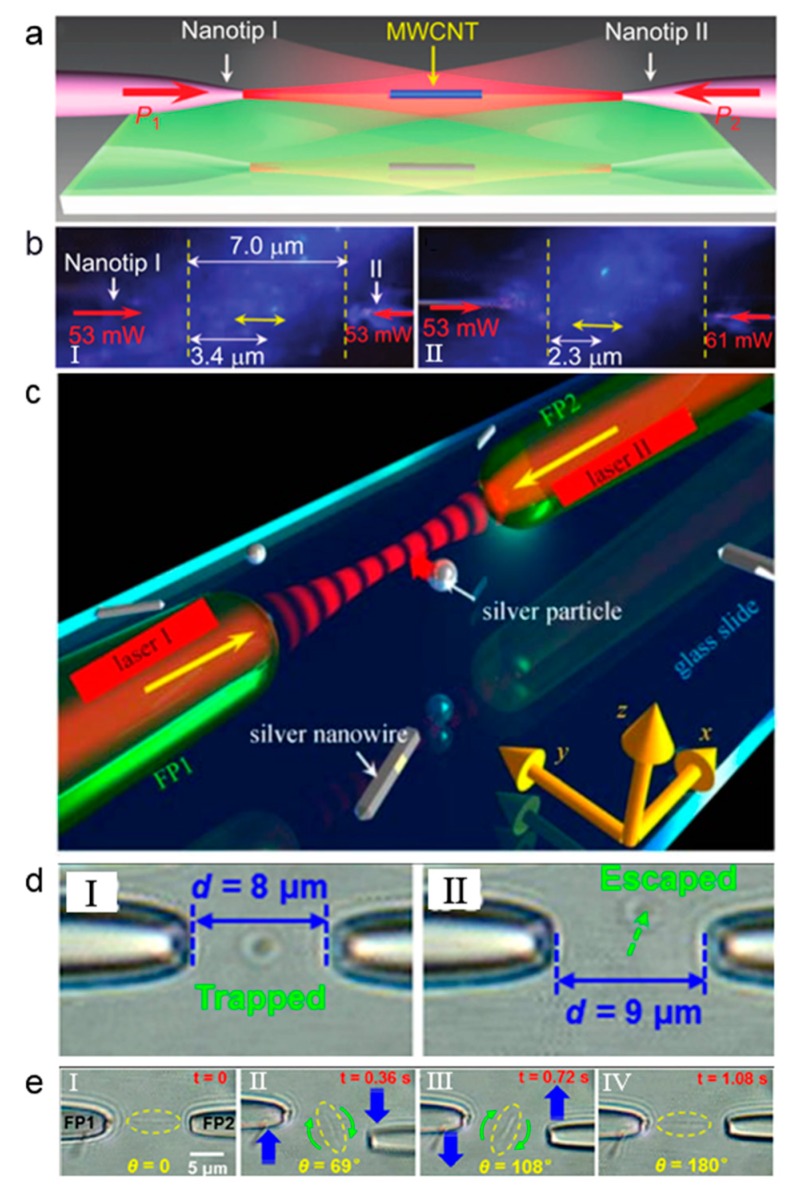
(**a**) Schematic depiction of the orientation and displacement of a single multiwalled carbon nanotubes (MWCNT) controlled using a dual-fiber-nanotip method. (**b**) Dark-field optical microscope images of MWCNT at different output power. Adapted with permission from Xin et al. [34]. (**c**) Schematic depiction of three-dimensional (3D) optical trapping of silver nanostructures. (**d**) Optical traps images at different distances. (**e**) Continuous microscopic images of silver nanowires trapped by rotation. Adapted with permission from Xu et al. [35].

The use of dual fiber tweezers also allows for more precise control of the nanoscale particles in addition to the manipulation of particles and cells with a relatively large size (micrometer scale). Figure 3a shows the schematic depiction of a dual-fiber-nanotip method for the manipulation of multiwalled carbon nanotubes (MWCNTs) with an outer diameter of only 50 nm and a length of 0.9 μm. The position and orientation of the MWCNT can be adjusted by changing the distance between the two nanotips and the input power of each nanotip. When *P*_1_ (optical power from the left fiber) remains unchanged and *P*_2_ (optical power from the right fiber) power increases, the orientation and position of the MWCNT change accordingly [34]. Xu et al. realized the three-dimensional (3D) optical trapping of silver nanoparticles and nanowires while using dual focused coherent beams [35] (Figure 3c). As the distance *d* (the gap between two fiber probes, FPs) increases, the initial stable trap state of the silver nanowires (diameter: 330 nm, length: 2.1 μm) is destroyed and the transverse Poynting vector points outward, which causes the particle to escape (Figure 3d-I,II). The nanowires are rotated clockwise by increasing the transverse distance between FP1 and FP2 to form an asymmetric field distribution (Figure 3e). Additionally, Liu et al. proved that the inclined dual-fiber optical tweezers have the ability of 3D trapping, which is related to the inclination angle of the fiber [36]. The combination of 3D printed Fresnel lenses on dual fiber surface was reported to be an excellent method to increase the trapping efficiency and stability [37]. In this scenario, the trap stiffness is increased by a substantial factor of 35–50.

## 4. Single Optical Fiber Tweezers for Trapping and Single Cell Analysis

### 4.1. Single Optical Fiber Tweezers for Particle/Cell Trapping

**Figure 4:**
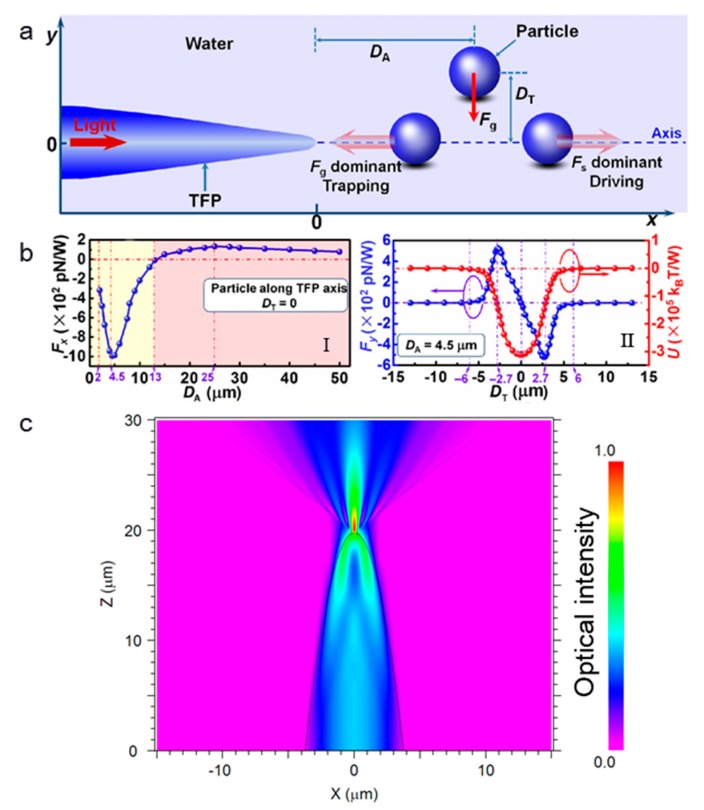
The principle for the single optical fiber tweezers trapping of particles. (**a**) Schematic illustration for particle trapping and manipulation with light launched into the tapered fibre probe (TFP). (**b**) Calculation result of the optical force: (Ι) axial force exerted on particles along the TFP axis as a function of *D_A_*, (II) transverse force (*F_y_*), and trapping potential (*U*) received by the particles at *D_A_* = 4.5 μm. Adapted with permission from Xin et al. [38]. (**c**) Optical intensity distribution near the fiber tip. Adapted with permission from Liu et al. [39].

Dual fiber tweezers still lack simplicity, so it is very important to realize the optical trapping and manipulation based on a single fiber, i.e., single optical fiber tweezers. In general, single optical fiber tweezers are tweezers that resulted from a single optical fiber. Figure 4a shows the working principle of particle trapping while using a single tapered fibre probe (TFP) [38], where *D*_A_ indicates the axial distance (*x* direction) of the particles to the tip of the TFP and *D*_T_ indicates the transverse distance (in *y* direction) of a particle to the axis. Once a laser beam is launched into the TFP, the light emerging from the TFP after focusing will exert an optical force on the particles. Optical force consists of two parts—the gradient force *F*_g_ and the scattering force *F*_s_. *F*_g_ tends to attract particles, while *F_s_* pushes away, and the resultant force of them together dominates the motion of the particles. The optical force on the particle can be calculated by integrating the Maxwell stress tensor around the particle. Figure 4b-I shows the calculated force *F_x_* exerted on the particle along the TFP axis as a function of *D*_A_ (i.e., *D*_T_ = 0). When the particle is close to the TFP tip (*D*_A_ < 13 μm), *F_x_* is negative, which means that there is a trapping force towards the tip. In this region, the gradient force *F*_g_ dominates the motion of particles and the particle can be trapped. When the particle is away from the TFP tip, *F_x_* is positive, which means that there is a driving force in the direction of light propagation. In this region, the scattering force *F*_s_ dominates the motion of particles and the particle can be pushed away. This different optical force-based manipulation mechanism results from the light distribution near the fiber tip. For a clearer description, Figure 4c shows the light distribution near the fiber tip [39]. It can be seen that the laser beam is focused at the fiber tip. At the tip of the fiber, the optical intensity reaches the highest and, therefore, there is a strong optical gradient force near the tip, which can be used for trapping. The gradient of intensity in the converging beam pulls small objects towards the focal point, while the radiation pressure tends to push them away along the optical axis. With gradient force being dominated, a particle can be trapped in three dimensions near the focal point by the optical gradient force [40].

The requirement to trap a particle is the light focusing at the fiber end, so that an optical gradient force can be exerted on the particles near the fiber end. Additionally, the main effects are the optical forces. The trapping mechanism for optical fiber tweezers is the same as that in COTs, both are resulted from the optical force (optical gradient force for trapping). For the COTs, light is focused by a high NA objective and the particle is trapped near the focus by optical gradient force. In single OFTs, light is focused by the end surface of the fiber and the optical gradient force also traps the particle. The ability of OFTs to trap particles highly depends on the shape of the end of the fiber, which directly affects the light focusing at the fiber end. For an optical fiber with a flat end surface, the light is difficult to focus and, thus, the particle is difficult to be trapped. Instead, it will be pushed away by the optical scattering force. For a tapered optical fiber with a parabolic or convex surface, light will be focused at the fiber end, and particles can be trapped by the generated optical gradient force. Light is highly focused near the fiber tip, as shown in Figure 4c. This highly focused light will generate a strong optical gradient force for particle trapping. Actually, Xin et al. calculated the trapping efficiency of a 10-µm polystyrene particle in both OFTs and COTs [38]. The calculated efficiency is 0.227 and 0.12 for OFTs and COTs, respectively. That means that the trapping efficiency for OFTs is not weaker than COTs. The main difference between the FOTs and the COTs is that the COTs realize a truly non-contact trapping, while particle might be contacted with the fiber surface after trapping in OFTs. By changing the shape of tapered fiber end so that the focal point is a few microns away from the tip of the optical fiber, we can realize a non-contact optical trapping [41]. The fundamental limitation for the trapping is the diffraction limit. For particles within the diffraction limit, it is difficult to trap.

**Figure 5:**
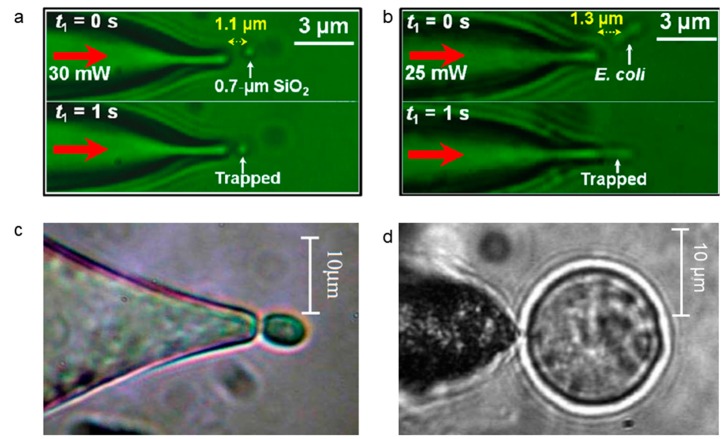
Single optical fiber tweezers are able to trap particles and cells with different sizes. Optical trapping of (**a**) a 0.7-µm-diameter SiO_2_ particles. Adapted with permission from Xin et al. [38], (**b**) *E. coli*. Adapted with permission from Xin et al. [38], (**c**) yeast cell. Adapted with permission from Grier et al. [42], (**d**) Chinese hamster ovary cell. Adapted with permission from Mohanty et al. [43].

Optical fiber tweezers can be used to trap particles and cells of different sizes, which range from few hundreds of nanometers to tens of micrometers. Figure 5 shows the optical trapping of different particles/cells from small to large. The main influence factor is Brownian motion and diffraction limit for the trapping of small particles. As shown in Figure 5a, the SiO_2_ sphere was 1.1 μm away from the optical fiber tip at *t*_1_ = 0 *s*, and it was trapped at the tip at *t*_1_ = 1 *s*. Likewise, *Escherichia coli* (*E. Coli*) was 1.3 μm away from the optical fiber tip at *t*_1_ = 0 *s*, and trapped at the tip at *t*_1_ = 1 *s* [38]. Figure 5c shows a tapered optical fiber with abruptly tapered twin-core, which is fabricated by fusing and drawing the twin-core fiber [42]. The twin-core fiber extends the functionality of the fiber optical tweezers, and allows for the particle trapping with orientation. The single optical fiber tweezers can even be used for the trapping of cells larger than 10 μm. Figure 5d shows the trapping of a mammalian cell with a size of about 15 μm [43].

**Figure 6:**
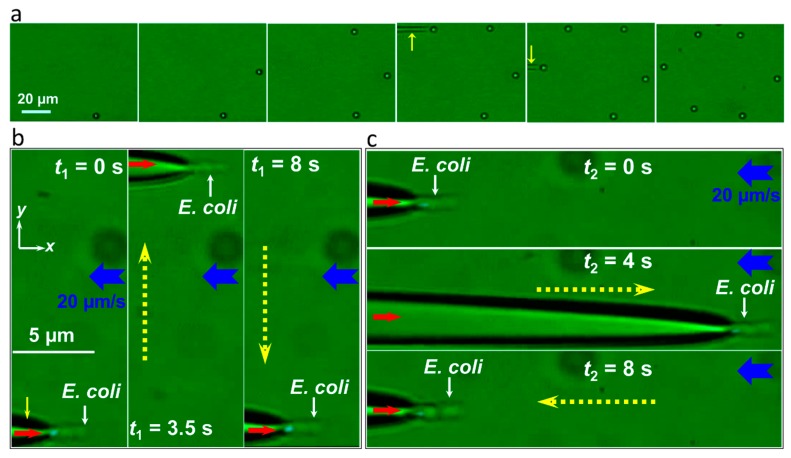
Images of using OFTs for flexible manipulation of trapped particles and bacteria. (**a**) Arrangement of six particles into a hexagon. The particles are picked up and placed into the designated postion after trapping by flexible moving the fiber (yellow arrow indicated). Adapted with permission from Xin et al. [38]. (**b**,**c**) Flexible manipulation of a trapped *E. coli* bacterium in flowing environment. The yellow solid arrow indicates the fiber, the blue arrows indicate the flowing direction. Adapted with permission from Xin et al. [44].

Single OFTs are widely used for optical trapping and manipulation due to the easy fabrication and simple structure. Without the need of high-NA objective for light focusing and the optical elements for laser beam expanding and light steering that are necessary in COTs, the OFTs have the advantages of compactness and high manipulation flexibility. By simply moving the fiber, the trapped particles can be flexibly manipulated accordingly. Figure 6 shows some examples for the flexible manipulation and moving of trapped particles and bacteria [38,44]. By simply moving the fiber, the trapped particles can be manipulated in three dimensions flexibly. Such manipulation enables the pick-up action for further precise arrangement of particles into designed patterns (Figure 6a). The manipulation can also be applied in flowing environment (Figure 6b,c) [44]. In addition, a single optical fiber can be inserted into the sample with any angles and any depth, which greatly increases the manipulation flexibility. Therefore, the single optical fiber tweezers can be used in many different environments, where COTs cannot be achieved. By cooperating with aspheric lens, optical trapping, and cooling of particles in vacuum has also been demonstrated [45]. For a direct comparison, Table 1 shows some features of single OFTs when compared with COTs.

### 4.2. Single Optical Fiber Tweezers for Cell Analysis

**Figure 7:**
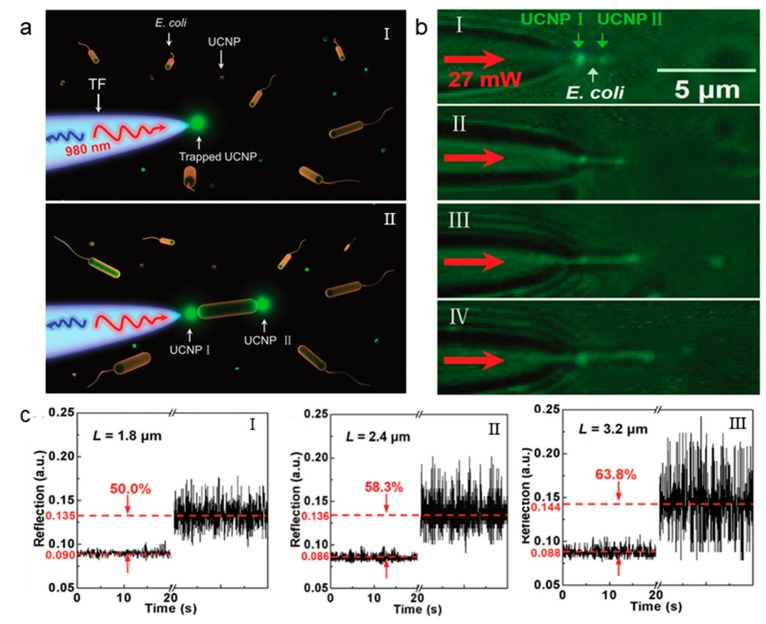
(**a**) Schematic and (**b**) experimental images of bacterial cotrapping and labelling. (**c**) Real-time reflected optical signal from the trapping and labeling of single bacteria with different sizes. Adapted with permission from Xin et al. [46].

In addition to the trapping of particles and cells, the single optical fiber tweezers are also widely used for single cell labelling and analysis after the cell is trapped. For example, a single bacterium can be labelled via the cotrapping of a single bacterium and upconversion nanoparticles (UCNPs) [46]. Figure 7a,b provide a clear description of how a tapered fiber probe (TF) traps a single UCNP and briefly labels the bacterium. A single UCNP (≈120 nm) undergoing Brownian motion is trapped at the tip of the TF, with a 980 nm wavelength laser beam. It is difficult to observe uncaptured UCNPs because of the small size of UCNPs. When are UCNPs is trapped to the tip, it emits green light excited by the 980 nm laser and could, therefore, be directly observed. After the trapping of the UCNP, a bacterium can be further trapped, and another UCNP can be cotrapped at the end face of the bacterium. The emitting green light provides a direct way for the labeling and observation of a single bacterium that is otherwise impossible in dark environments. Figure 7b shows the images of labelled individual bacteria with different lengths. The labelling also allows for the single bacterium analysis via optical signals. Figure 7c shows the reflection signal of labeled bacteria with lengths of 1.8, 2.4, and 3.2 μm, respectively.

**Figure 8:**
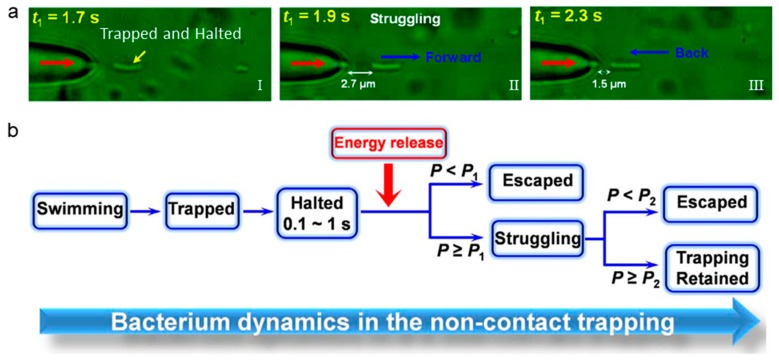
Dynamic analysis of motile bacteria via non-contact optical trapping of single bacteria. (**a**) Optical microscope images capture and struggle with the process of *E. coli* bacterium. (**b**) Schematic of the bacterium dynamics in the non-contact trapping. Adapted with permission from Xin et al. [41].

In addition to the analysis of single bacteria from the optical signals, optical trapping via single OFTs also allows for the energy analysis of motile bacteria. Xin et al. used a modified tapered fiber to trap *E. coli* in solution and studied the kinematics of single bacteria [41]. Figure 8a shows the dynamic of a motile bacterium in the trapping potential. At *t*_1_ = 1.7 s, the motile bacterium was stably trapped at a distance of 1.9 μm away from the fiber tip and kept halted for about 0.2 s (Figure 8a-I). At *t*_1_ = 1.9 s, the bacteria began to struggle in the trapping potential after the stored energy release (Figure 8a-II). Later, it was trapped back toward the fiber (Figure 8a-II). At *t*_1_ = 2.3 s, the bacterium was trapped back with a gap of 1.5 μm to the fiber tip. Figure 8b can analyze and explain the phenomenon of bacteria back-and-forth. In solution, the motile bacterium is swimming. When near the fiber tip, it was first trapped by the OFTs, and then halted for about 0.1–1 s. After that, the energy that was stored in the bacterium was released and converted into kinetic energy. If the trapping potential is smaller than bacterium energy, the bacterium can be released. Otherwise, the bacterium can be struggling in the trapping potential. Further, the bacterium can further escape from the trap if the stored energy is further released, and is larger than the trapping potential. Otherwise, the bacterium can be constantly trapped. This method provides a simple method for the analysis of bacterium dynamics. 

## 5. Optical Fiber Tweezers for Cell Assembly

In addition to the trapping and manipulation of a single particle, OFTs can also be used for the cell assembly, which is very important for the study of cell-cell interaction and communication. In addition, the assembly of randomly distributed cells into regular shaped structures and arrays also plays an important role in many other biomedical and bio-optical fields, such as tissue engineering [47], drug delivery, and targeted therapy [48,49].

### 5.1. Cell Assembly by the Optical Binding

**Figure 9:**
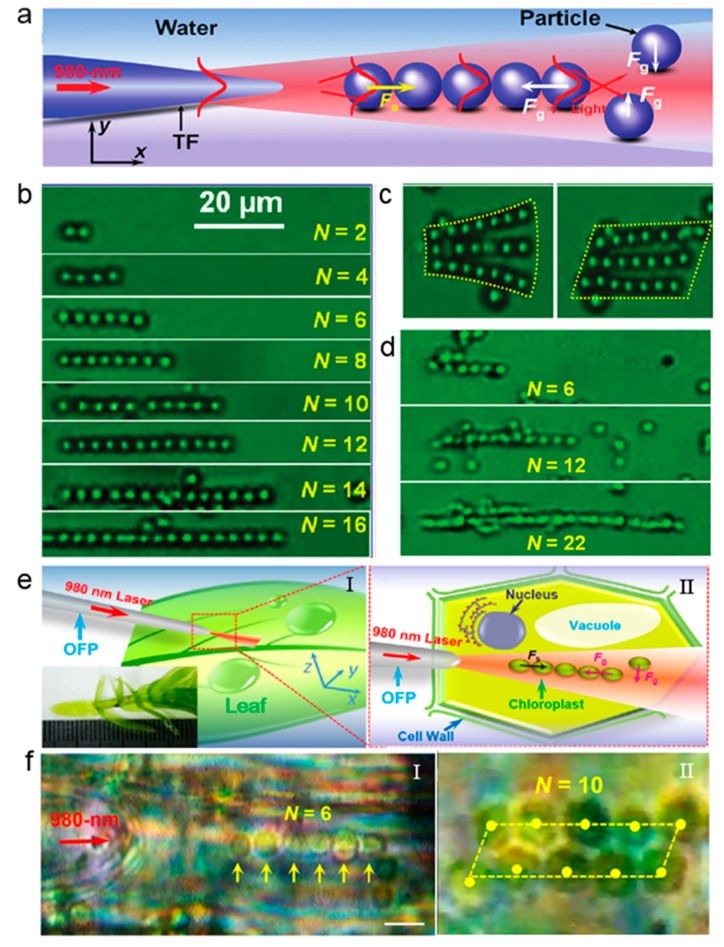
(**a**) Schematic of optical assembly of particles and cells via optical binding. Microscopic images of (**b**) one-dimensional (1D) patterned particle chains and (**c**) two-dimensional (2D) patterning of particle arrays. (**d**) Microscopic images of yeast cell chains. Adapted with permission from Xin et al. [51]. (**e**-**Ι**) Schematic of an optical fiber probe (OFP) which is aslant placed above the plant leaf with a laser at 980 nm launched. The inset shows a living plant (*Hydrilla verticillata*) on a glass slide. (**e**-**II**) Schematic of optical binding of chloroplasts inside a plant cell, which shows a row of chloroplasts confined in the optical axis and bound to each other, resulting from the cooperation of *F*_g_ and *F*_s_. (**f**) Microscopic images of the arranged chloroplast chain and rectangle. Adapted with permission from Li et al. [52].

Tam et al. used multiple optical fibers to form dense optical wells to capture and arrange parallel microspheres [50]. However, this approach is too cumbersome and it would be nice to capture and arrange particles with a single fiber. Figure 9a schematically shows the mechanism of particle chain formation while using a single optical fiber probe (TF) [51]. When a 980 nm laser beam is launched into the TF, the resulting optical force will act on the particles. Firstly, the scattering force will drive the first particle away along the TF axis. Subsequently, the particles beside the TF axis and near the first particle can be trapped to the TF axis resulting from transverse gradient force. Under the action of axial gradient force of the tail particle, the particle is closely bound to the former and, therefore, multiple particles and cells are assembled together by the cooperation of optical scattering force and gradient force. By using the above principle, the particles can be arranged into a one-dimensional particle chain (Figure 9b) or even a two-dimensional graphic particle array (Figure 9c). The binding ability also works on cells in the same manner. Figure 9d shows the yeast cell chain formed by optical binding. This method can even be used for the assembly of organelles inside a living cell. For example, Li et al. investigated the noncontact intracellular binding and controlled manipulation of chloroplasts in vivo while using optical fiber probes [52]. Figure 9e shows the optical binding of chloroplasts in plant cells using optical fiber probe (OFP), which is above a plant leaf with an approximately 3-μm gap between the fiber tip and the leaf. Figure 9f shows the arranged one-dimensional (1D) and two-dimensional (2D) chloroplast structures. 

### 5.2. Cell Assembly by Extended Optical Gradient Force

**Figure 10:**
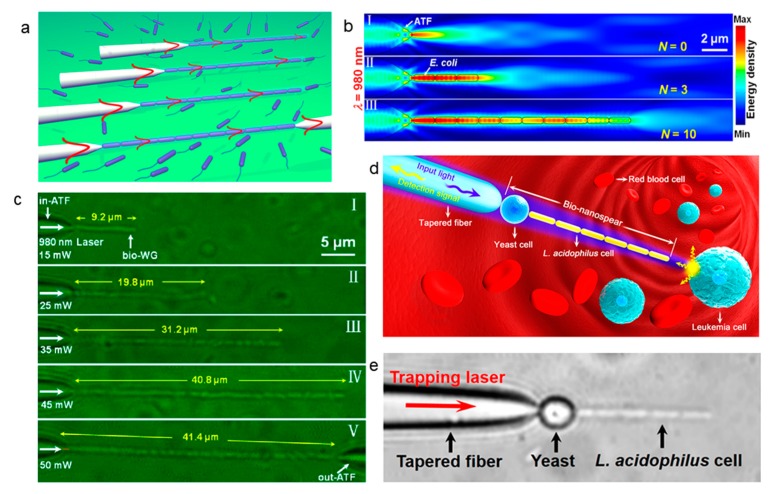
Optical assembly via extended optical gradient force. (**a**) Schematic of optical assembly and biological optical waveguides (bio-WGs) formation. (**b**) The distribution of energy density in ATF when different numbers of *E. coli* were captured (**c**) Images of formed bio-WGs with different lengths. Adapted with permission from Xin et al. [53]. (**d**) Schematic illustration of the assembled bio-nanospear. (**e**) Optical image of the bio-nanospear assembled from a yeast and *L. acidophilus* cells. Adapted with permission from Li et al. [54].

Multiple cells can also be assembled solely by optical gradient force in addition to the cooperation between optical scattering force and gradient force for cell assembly. Xin et al. reported an extended optical gradient force-based method for cell assembly [53]. In general, a cell is first trapped at the tip of an ATF (abrupt tapered optical fiber) by optical gradient force, light can propagate along the cell, and refocused at the cell end surface, as shown in Figure 10a. This refocused light can generate an optical gradient force on other cells. In this regard, the optical gradient force is extended. Multiple cells are then assembled via the extended optical gradient force, and light can propagate along the multiple cells, Figure 10b shows the energy density distribution of the captured *E. coli* of (I) *N* = 0, (II) *N* = 3, (III) *N* = 10. It is can be seen that light is highly concentrated at the tip of the fiber, which provides a higher trapping efficiency to *E. coli*. Using this phenomenon, Xin et al. constructed biological optical waveguides (bio-WGs) based on *E. coli* bacteria, as shown in Figure 10c. This all cell-based bio-WGs opens the door for the fabrication of biological waveguides using cells. Because all of the materials of the bio-WGs are biological cells, these waveguides are highly biocompatible, when compared with conventional optical waveguides that are based on silica and other organic/inorganic materials. Based on this idea, Li et al. made a bio-nanospear in which the “handle” was made of a tapered fiber and the “head” was assembled with a yeast cell and a chain of nanosized *Lactobacillus acidophilus* (*L. acidophilus*) cells [54], as schematically shown in Figure 10d. At first, an 808 nm laser beam was launched into the tapered fiber to trap a yeast cell at the tip and then to trap a *L. acidophilus* behind the yeast. The spherical yeast focused the laser beam and exerted a strong optical force on the *L. acidophilus* cell that was trapped behind the yeast. Figure 10e is the image of the biological spear. With precise manipulation, the formed bio-nanospear can guide the input light to a specific location and detect optical signals from biological cells, such as individual leukemia cells in human blood. In addition, the group stably trapped a bio-microlens (yeast or a human cell) on a compact fiber probe by optical force, the excitation light was, therefore, confined in a subwavelength region, which resulted in the enhanced up-conversion fluorescence [55]. 

### 5.3. Cell Separation after Assembly

**Figure 11:**
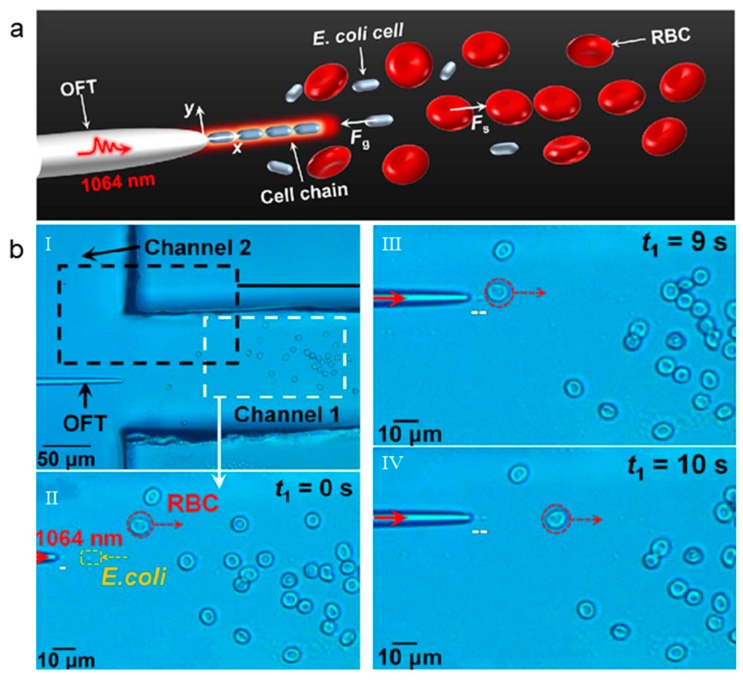
(**a**) Schematic of *E. coli* trapping and arrangement at the tip of the OFTs with red blood cells (RBCs) pushed away. (**b**-**I**) Laser OFF. In channel 1, the *E. coli* and RBCs are mixed together. (**b**-**II**–**IV**) Laser ON. The *E. coli* is attracted and the RBCs are released. Adapted with permission from Liu et al. [56].

The separation and screening of particles can also be realized by OFTs after cell assembly. Liu et al. proposed a compact, miniaturized optofluidic chip integrated with OFT in a T-type microfluidic channel [56] (Figure 11a) for the selective capture of cells and bacteria. It was verified that the OFTs can capture and assemble *E. coli* cells, and it can drive red blood cells (RBCs), to experimentally verify the OFTs' selective capture capability. It is reported that the focusing characteristics of the fiber tip will change with its shape, which results in different optical forces. Taking advantage of this feature, a special fiber tip was used to provide push force to the red blood cells and trapping force to the *E. coli* cells, thus achieving cell sorting. Figure 11b experimentally verified the OFTs' selective trapping, which realized the trapping and delivery of *E. coli* and the push of RBCs. When the laser was turned off, the *E. coli* and RBCs were mixed together in the channel 1. Once laser was on, the *E. coli* were trapped and assembled, while the RBCs were immediately pushed away (Figure 11b-I). Finally, the trapped and assembled *E. coli* were sent to channel 2, while the RBCs were left in channel 1, achieving the 100% pure separation (Figure 11b-II–IV).

## 6. Structured Optical Fiber for Trapping and Manipulation

The trapping and manipulation of different objects make a high requirement on structured OFTs, although single OFTs have been successfully used in many studies [57,58,59]. These structured OFTs will provide a new platform for optical manipulation, especially for the trapping of nanosized particles that need to overcome the diffraction limit. Structured OFTs will serve as a new candidate with much more powerful trapping and manipulation capabilities [4,60,61].

### 6.1. Internal Structured Optical Fiber Tweezers for Non-contact Trapping

#### 6.1.1. Fiber-based Total Internal Refection Lens

**Figure 12:**
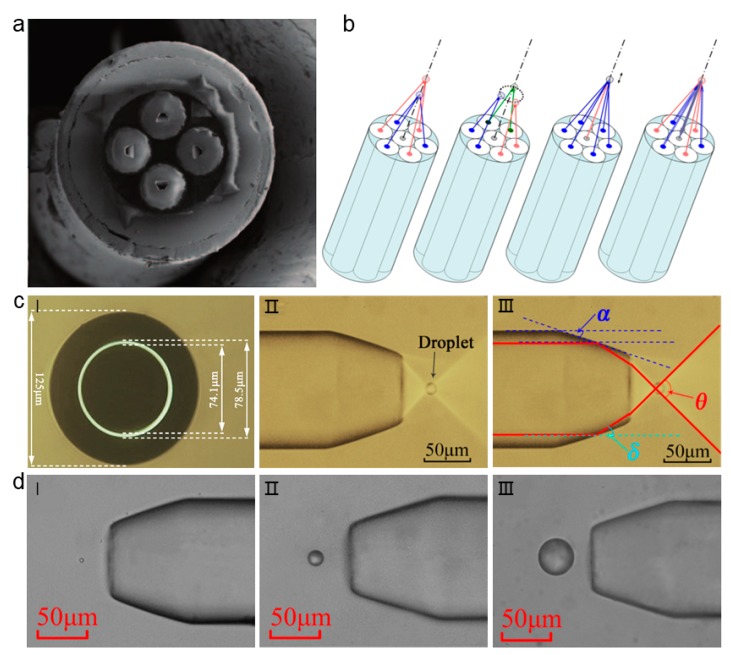
Fiber-based total internal refection lens for non-contact trapping. (**a**) Scanning electron microscope (SEM) image of the optical fiber probe. The gradient inclined hole is obtained by shaping the end of the fiber. (**b**) Different functions can be achieved by using optical fiber bundles. Adapted with permission from Liberale et al. [62]. (**c**-**I**) The profile view of the annular core fiber, the external diameter of annular fiber is 78.5 μm while the internal diameter is 74.1 μm, and the surrounding cladding diameter is 125 μm. (**c**-**II**) The lateral view of the annular core fiber. (**c**-**III**) Schematic diagram of the optical path. (**d**) Images of oil droplets captured by the annular core fiber optical tweezers. The diameters of the oil droplets are (**d**-**I**) 3.8 μm; (**d**-**II**) 16.4 μm; (**d**-**III**) 40.1 μm. Adapted with permission from Liu et al. [63].

There are many ways to achieve contactless capture, and the fiber-based total internal refection lens is one of them. In one case, a bundle of optical fiber was encapsulated in a quartz capillary and fixed with epoxy resin [62]. The end surface of optical fiber is properly processed to make the light propagation between the optical fiber and the surrounding medium in a total internal reflection form, so as to achieve the high numerical aperture focusing and realize the purpose of capture particles that are far away from the fiber end (Figure 12a). Moreover, the direction of light propagation is inclined to the z axis in this structure, and the exerted scattering force is therefore greatly weakened. A 3D optical trap that is constructed by the optical fiber with special internal structure can capture and manipulate objects in a non-contact and non-invasive manner. Different functions of this structure can be achieved and multiple potential wells can be implemented at different or same distances from the fiber. By changing the angle of the light, the trapped particles can be slightly moved when simultaneously collecting optical signals (Figure 12b).

In another method, an annular core fiber is used to make a new type of OFTs (Figure 12c-I) [63]. The end face of the annular core fiber is made into a special frustum cone shape, which can generate a strong optical force to capture droplets in a non-contact form. The light beam is completely reflected at the edge of the cone, and then deflected away from the end of the fiber. Therefore, objects can be captured and manipulated in a non-contact manner (Figure 12c-II,III). The special OFTs with a magnetic ring structure can capture a wide range of objects and droplets ranging from three microns to 40 microns in diameter. The shape of the trapped droplets can be perfectly maintained while using this non-contact method (Figure 12d).

#### 6.1.2. Graded-Index Lens

Graded-index fiber can also achieve the non-contact capture and manipulation of objects. Developing adjustable OFTs can improve the operating ability and range of applications, which is conductive to its application of optical tweezers in more fields [44,64]. For example, the combination of OFTs and microfluidics can realize the adjustable working distance of optical tweezers [65,66].

**Figure 13:**
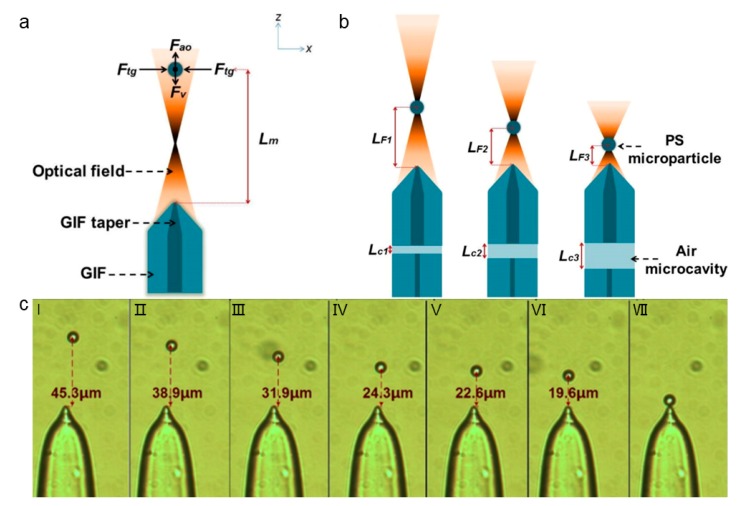
(**a**) Optofluidic tunable manipulation of microparticles by changing the laser power, flow rate and shape of the tapered fiber. (**b**) The operating range of OFTs can be adjusted by changing the length of the optical microcavity. The optical microcavity is the air cavity inside the fiber, as marked in aqua green and indicated by the dashed arrow. (**c**) Optofluidic tunable manipulation of microparticle with different working distance. Adapted with permission from Gong et al. [65].

Graded-index fiber taper has been proved to own a strong focus effect [66], and it enables optical trapping with adjustable working distances in liquid flow, combined with an optical microcavity (Figure 13). In this situation, the graded-index fiber and single-mode fiber are aligned in the capillary to form an air microcavity, the length of which can be adjusted by shifting the platform, and then directly affects the focusing ability. By adjusting the light focus or the balance between optical force and drag force, the adjustable OFTs are realized while improving the working range and operating ability (Figure 13b). The tip of graded-index fiber has lower transmission loss and the stronger transverse gradient force, which can enhance the operating range of OFTs. The graded-index OFTs are adjustable for non-contact trapping of particles with controllable working distance by changing the input power of light, flow rates, length of the micro-cavity, and other factors (Figure 13c). 

### 6.2. Surface Structured OFTs for Nanomanipulation

The latest demand for nanotechnology is that it can precisely and noninvasively manipulate nanoscale objects [67]. However, trapping of objects with nanometer scale is of great challenge due to the diffraction limit and the strong Brownian motion [68,69]. Therefore, the nanomanipulation of OFTs has become the focus of researcher's attention [70]. Until now, different OFTs-based nanomanipulation techniques have been developed, such as plasmonic nanostructure-based, photonic nanojet-based, and connected fiber/nanoject combined forms.

#### 6.2.1. Plasmonic Nanostructure-based Fiber Tweezers

**Figure 14:**
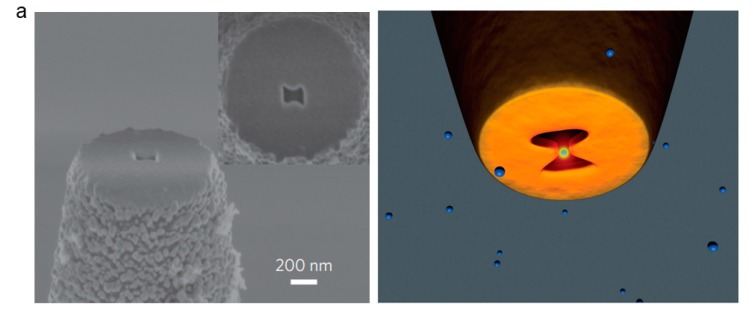
(**a**) SEM image of surface structured fiber with a plasmonic bowtie aperture. Adapted with permission from Pang et al. [71]. (**b**) Structure diagram for nanoparticle trapping on the bowtie nano-aperture structure.

A plasmonic nanostructured-based fiber tweezers can be used for nanomanipulation. In this case, a metal coating is applied to the tapered fiber and a bowtie plasmonic aperture is designed to capture and manipulate the nanoparticles (Figure 14a) [71]. The plasmonic nanostructures at the fiber end surface plays the key role in this technique, providing a powerful way of dealing with individual atoms that are attached to surfaces. The trapping mechanism of this optical fiber structure is based on the self-induction back-action, which can reduce the influence of the photothermal effect on the captured object [72]. The trapped specimens play an active role in the trapping mechanism, and the required field strength is several orders of magnitude weaker than traditional optical fiber tweezers. This technique works as a scanning optical microscope probe and nano-optical tweezers for nanomanipulation in three dimensions. The end of the probe is used to manipulate and detect objects, while the object is captured and detected while using optical fibers [70,73,74]. Using this plasmonic nanostructured fiber tweezers, sub-100-nm particles can be trapped and manipulated in three dimensions (Figure 14b). 

#### 6.2.2. Photonic Nanojet-based Fiber Tweezers

**Figure 15:**
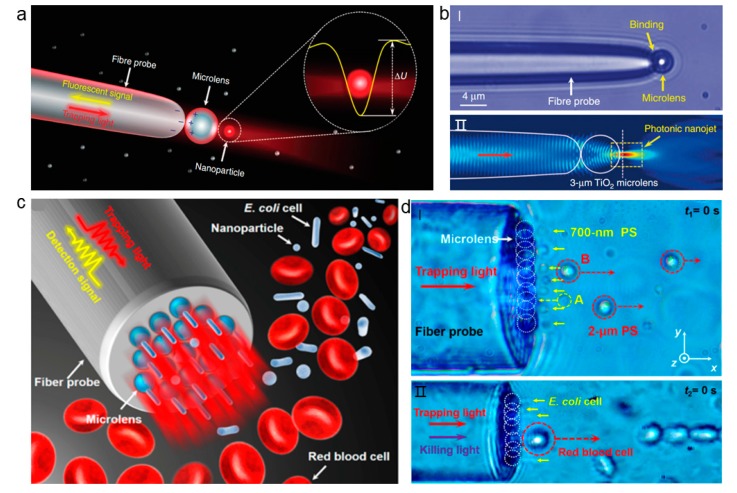
Photonic nanojet-based fiber tweezers. (**a**) Schematic of photonic nanojet-based trapping of nanoparticles. (**b**) Microscopic image (**I**) and simulation results of the photonic nanojet (**II**). Adapted with permission from Li et al. [81]. (**c**) Schematic of a parallel photonic nanojet array for selective trapping and manipulating of nanoparticles and cells in blood solution. (**d**) Images for photonic nanojet array trapping and separation of different particles and cells. Adapted with permission from Li et al. [82].

Currently, the selective capture of nanoscale targets and the simultaneous capture of multiple targets are facing huge challenges [67,75]. With high focusing characteristics, photonic nanojet can effectively reduce the critical size of the captured object [76,77]. Since being first reported by Chen et al. [78], photonic nanojets have been applied in many fields [79,80]. These photonic nanojets can be used for optical trapping and nanomanipulation when integrated into a fiber end. In this case, a microsphere that is positively charged is attached to the end of a tapered optical fiber (Figure 15a) [35]. Light is highly focused at the end of the sphere due to the photonic nanoject mechanism of the microsphere (Figure 15b). This light focusing can exert enhanced optical force on the nanoobject, and the shadow of the photonic nanojet surface forms a potential well for capturing nanoparticles in a non-contact way (Figure 15a). Therefore, this structure can be used for the trap of nanoparticles as well as biomolecules, such as DNA. Combining the three-dimensional optical manipulation with signal enhancement, this technique can also be used for the detection of nanoparticles and biomolecules.

In addition, multiple dielectric microparticles can be arranged onto the surface of a fiber end to form photonic nanojet array [82]. The back of microparticles produces a highly focused beam because of the high refractive index and low light absorption of dielectric particles, which serves as photonic nanojet array, and can thus manipulate and detect nanoparticles and subwavelength cells. The photonic nanojet array assembled with microspheres can form tens to hundreds of nanowells and then manipulate and detect multiple nanoparticles or subwavelength cells simultaneously at low power (Figure 15c). The device owns a strong capture ability, while the optical power is far lower than the COTs, it therefore will not easily generate a photothermal effect. In addition, the optical signal that is transmitted through the front end of the optical fiber can also be detected through the same fiber in reflection. This can be used for the detection of different particles and cells. For different cells, the exerted force is different due to the different sizes. Scattering forces cause the large particles to move in the direction of light propagation, while the smaller bacteria cells can be trapped. This can be used for the selective trapping and separation of different cells (Figure 15d).

#### 6.2.3. Connected Fiber/Nanojet Combined Fiber Tweezers

**Figure 16:**
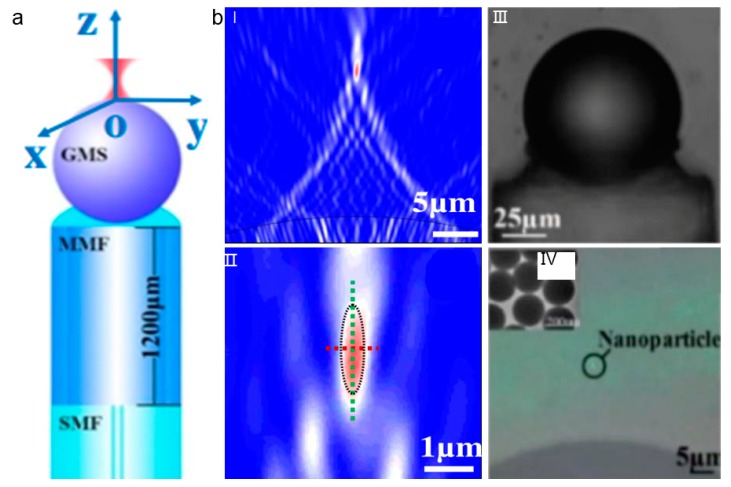
(**a**) Schematic of the fiber probe with a glass microsphere (GMS) attached. (**b**) (**I**),(**II**)Simulation results of light distribution; (**III**) Microscopic image of the probe. (**IV**) Optical trapping of nanoparticles with diameters of 200 nm. Adapted with permission from Tang et al. [83].

The connected fiber with combined nanojet at the surface can also be used for the manipulation of nanoparticles in addition to the solely surface modification of optical fiber end [83]. In this case, the single-mode fiber (SMF) and multi-mode fiber (MMF) are connected together to generate Bessel beam, which produces a narrow output laser, and a glass microsphere (GMS) is attached to the surface of the MMF to form a photonic nanojet (Figure 16a). At the tip of the optical fiber, the narrow laser beam is further focused into a nanoscale point by the glass spheres at high magnification (Figure 16b). This technique can thus be used for the trapping and manipulation of nanoparticles in a non-contact manner.

## 7. Subwavelength Optical Fiber Wire for Evanescent Fields-based Trapping and Delivery

Optical tweezers capture and manipulate particles with the optical force that is generated by a highly focused laser [84]. The diffraction limit and focusing depth of optical tweezers in space limit the applications of optical tweezers in the manipulation of particles and continuous delivery of particles in a long range. This problem can be solved when using a subwavelength optical fiber wire with evanescent fields at the surface, which can capture and manipulate particles in a long range [85]. Similar to the optical waveguides, optical forces that are generated by the evanescent field around the subwavelength fiber wire can also be used for the capture and delivery of particles [86,87,88].

**Figure 17:**
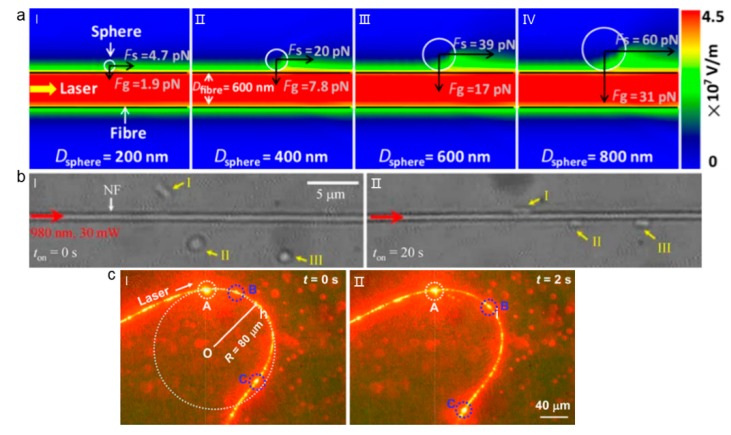
Evanescent field-based capture and delivery along optical fiber wire. (**a**) Simulation distribution of evanescent wave field around a fiber wire with different sized particles trapped. Adapted with permission from Xu et al. [90]. (**b**) Trapping of *E.coli* along an optical fiber wire. Adapted with permission from Xin et al. [91]. (**c**) Delivery of nanoparticles along bent optical fiber wire. Adapted with permission from Li et al. [92].

For the subwavelength optical fiber wire, as shown in Figure 17a, near the optical fiber surface, evanescent fields decay exponentially away from the surface. At the fiber surface, the optical intensity is the maximum, and field gradient exists along the direction of the exponential decay, which is perpendicular to the fiber wire. With this field gradient, optical gradient force is generated perpendicular to the fiber surface, which can be used for particle trapping. Figure 17a also shows the calculated optical gradient force. Accordingly, the particle is stably trapped at the fiber surface by the optical gradient force. Along the fiber, there is an optical scattering force, which delivers the particles along the fiber surface in direction of light propagation [89]. Size dependent trapping and delivery of polystyrene spheres was achieved while using a 600 nm diameter fiber wire (Figure 17a). The optical force exerted on the polystyrene sphere increased with the increase of the diameter, and the delivery speed of the larger sphere was also higher than that of the smaller sphere. At low input laser power, the larger sphere was more likely to be captured on the surface of the fiber and then delivered along the propagation direction [90]. This subwavelength optical fiber wire can also be used for the manipulation of biological cells in addition to the manipulation and delivery of particles. For example, *E.coli* can be stably trapped on the surface of the fiber by optical gradient forces (Figure 17b) [91], and the trapped *E. coli* can further be delivered along the fiber, even in microfluidic environment.

In addition to the delivery in a straight trajectory along a straight fiber wire, particles can also be delivered along a bent optical fiber wire. For example, Li et al. demonstrated the delivery of nanoparticles along an arbitrary bent fiber wire (Figure 17c) [92]. The relationship between bending loss, bending radius, and center angle were studied. For a specific input power, the light capture and transmission had a corresponding minimum bending radius. The delivery of nanoparticles was first demonstrated by using 650 nm red light propagating along a 600-nm diameter optical fiber wire. The nanoparticles can be captured by the optical gradient force when the power of incident light was increased to 12 mW, and then delivered along the bent fiber in the direction of light propagation direction by the scattering force caused by evanescent field.

**Figure 18:**
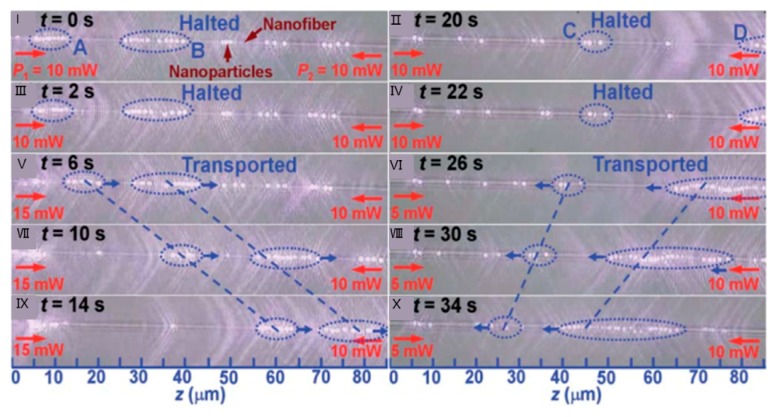
Bidirectional transportation and controllable positioning of nanoparticles. Adapted with permission from Lei et al. [93].

In addition to the delivery of particles in one direction along the fibre, bidirectional manipulation of particles can also be realized while using optical fiber wire [93,94]. In this case, two laser beams were transmitted in reverse direction into the fiber to realize bidirectional manipulation of particles. The magnitude and direction of the scattering force can be changed to control the delivery direction of the particles by changing the laser power of the input fiber (Figure 18) [82].

## 8. Optical Fiber-based Massive Photothermal Assembly

The photothermal effect due to light absorption can also be used for the trapping and manipulation of particles, in addition to the trapping and manipulation of particles by optical force using optical fiber tweezers. The light absorption of surrounding medium and particles can induce different photothermal effects. For the medium absorption, the main effect is the thermophoresis, where the temperature gradient that is induced by the light absorption can result in the particle migration responsive to temperature gradient by the Soret effect [95]. While for the particle absorption, photophoresis will induce the particle migration. The uneven photothermal phenomenon of light-irradiated particles can keep the high-absorptive particles away from the light-induced hot point [96,97], while the low-absorptive particles tend to the hot point, and the photothermal effect can, therefore, manipulate the large-scale particles [98]. 

For the photophoresis, one of the important parameters is the asymmetrical factor of heat distribution *J*, which can directly represent the heat flow [97]. The *J* factor is given by:

(7) $$J=\frac{6\pi n_{p}\kappa_{p}}{n_{f}^{2}R^{3}\lambda }\cdot \int_{0}^{R}\int_{0}^{\pi }\frac{{\left|E\left(r,\theta \right)\right|}^{2}}{{\left|E_{0}\right|}^{2}}r^{3}cos\theta sin\theta \cdot d\theta \cdot dr$$

where $n_{p}$ is the refractive index of the particle, $\kappa_{p}$ is the absorption rate of the particle, $n_{f}$ is the refractive index of the solution, *R* is the radius of the particle, λ is the wavelength of incident light, E(r,θ) is the local electric field intensity within the particle, and $E_{0}$ is the electric field intensity of incident light. For particles in the liquid medium, the expression formula of the photophoretic velocity $V_{P}$ is expressed, as follows [97]:

(8) VP=−βTAr0218μv0kfIJln3+4(ln3−1)LSr0(kpkf+2)(1+2LSR)

where $\beta_{T}$ is the thermal expansion coefficient of solvent, *A* is Hamaker constant, $r_{0}$ is the radius of the solvent molecules, and μ is the viscous coefficient of liquid, $v_{0}$ is the solvent the characteristics of the molecular volume, $k_{f}$ is the thermal conductivity solvent, *I* is light intensity, $L_{S}$ is particle relative to the slip length of water, $k_{p}$ is the thermal conductivity particles, and *R* is the particle radius. For a given solution and a particle of the same material, the photophoretic velocity $V_{P}$ can be expressed, as follows (*C* is a constant, C=−βTAr0218μv0kfln3+4(ln3−1)LSr0(kpkf+2)) [99]:

(9) VP=CIJ(1+2LSR)

**Figure 19:**
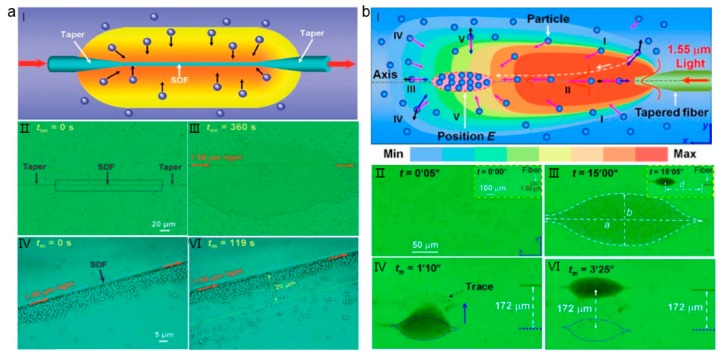
(**a**) Massive photothermal assembly and migration of particles and *E.coli* using subwavelength diameter fiber. (**b**) Assembly and migration of particles using a tapered optical fiber. Adapted with permission from Xin et al. [98].

In many situations, the photothermal effect is the combination of both the thermophoresis and the photophoresis. When compared with optical force, the force that is induced by photothermal effect is much stronger and, thus, can be used for massive particle trapping and assembly. For example, Lei et al. demonstrated the photothermal assembly of particles and *E. coli* while using a subwavelength diameter fiber (SDF) [100]. When the 1550 nm laser is launched into the fiber, the leaking light radiates the object and creates a negative photophoresis that pulls the object toward the fiber. The SDF functions as a linear “light source” and particles or other small objects around the SDF are exposed to the radiation. The energy of the leaking light will be absorbed by the particles and converted into a local heat distribution. The uneven heat distribution inside each sample causes the photothermal effect to drive the particles (Figure 19a). When suspended in medium, the particles will also move along the temperature gradient, usually from hot to cold. Particles move towards the strong region and gather in the center of the region, where the negative photophorsis force and the temperature gradient force are balanced, due to the combination effect of the photophoresis and thermophoresis. By moving the SDF, the assembled particles and bacteria can be migrated along the moving direction. Alternatively, a tapered optical fiber can also be used for the massive photothermal assembly of particles [98]. As shown in Figure 19b, particles can be assembled to the end of the fiber with a distance of several tens of microns due to the photothermal effect. By moving the fiber, the assembled particles can be migrated with an efficiency up to 95%.

## 9. Opto-Thermophoretic Fiber Tweezers

**Figure 20:**
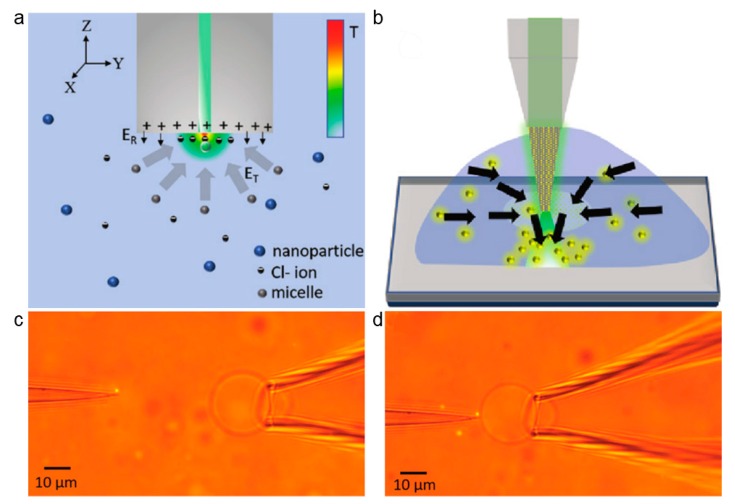
Opto-thermophoretic fiber tweezer (OTFT) for nanoparticle trapping and manipulation. Adapted with permission from Kotnala et al. [105]. (**a**) Schematic of OTFT, opto-thermoelectric force is used for nanoparticle trapping. (**b**) Schematic for nanoparticle assembly and concentration using OTFT. (**c**) and (**d**) OTFT serves as a nanopipette for single nanoparticle delivery.

In addition to the optical force and photothermal effect, the electric fields can also contribute to the trapping and manipulation [101,102,103,104]. Kotnala et al. demonstrated an opto-thermophoretic fiber tweezer (OTFT) [105], which combined the photothermal effect and electric field effect into the fiber tweezers. These fiber tweezers are a new strategy for the low power manipulation of nanoscale objects. In OTFT, a layer of porous gold film was deposited on the fiber tip to form a thermoplasmonic fiber tip, which converts photons into phonons. The charged molecules were adsorbed onto particle surface to form a charged molecular layer, which forms into positive micelles, and Cl^−^ molecules work as counterions. With light irradiation, the photothermal effect induced thermophoresis results in the migration of particles. The positive micelles and Cl^−^ ions were separated, inducing an electric field between the fiber tip and the particles due to the different Soret coefficient, which is given by [105]:

(10) $$E_{T}=\frac{k_{B}T▽T}{e}\frac{\sum_{i}Z_{i}n_{i}S_{Ti}}{\sum_{i}Z_{i}^{2}n_{i}}$$

where *i* is the ionic species, $k_{B}$ is the Boltzmann constant, *T* is the environmental temperature, ▽*T* is the temperature gradient, *e* is the elemental charge, $Z_{i}$ is the chrage number, $n_{i}$ is the concentration, and $S_{Ti}$ is the Soret coefficient of *i* species. An electric field *E*_T_ pointing toward the fiber tip is generated, due to the higher molecular mass and larger Soret coefficient of the positive micelle. The resulting thermo-electric field results into the trapping of the positively charged particles (Figure 20a). OTFT uses opto-thermoelectric force to capture particles, which have the unique advantages as compared with normal OFTs: it can be used to capture wide range of nanoparticles with low power consumption (less than 1 mW). By increasing the power, the increased convective flow and thermophoretic force can be used for particle assembly and concentration (Figure 20b, power: 10 mW). By manipulating the fiber, the trapped nanoparticles can also be used for multifunction applications. For example, it can be used for the delivery of a single nanoparticle to a lipid vesicle membrane (Figure 20c).

## 10. Conclusions

In conclusion, this review presents recent development of optical fiber-based tweezers for optical trapping and manipulation. Different types of OFTs were discussed, including their corresponding principles and applications. Single OFTs enable the targeted trapping and flexible manipulation of particles and cells while using optical force, and enable single cell analysis. Evanescent field from subwavelength optical fiber wire enables the long-range manipulation and delivery of different targets. The photothermal effect of different fiber forms can be used for massive assembly and migration. The opto-thermalphoretic fiber tweezers are able to multifunctional trapping and manipulation of nanoparticles with low power consumption when combing the electric field. Fiber optical tweezers will provide many new possibilities for different applications in the near future due to the advantages of easy fabrication, compact size, and flexible manipulation.

Although great progress has been made in OFTs, there are still many open challenges, but also opportunities. First, the direct contact between the sample and fiber end surface might induce mechanical damage on the samples. Therefore, the non-contact and damage-free trapping technique based on OFTs is necessary to develop. Second, stable trapping and flexible manipulation of samples with sizes in the nanometer scale to overcome the diffraction limit while using OFTs is still a big challenge. In particular, stable trapping of single biomolecules is very difficult, but will be of great importance for single molecular analysis. Third, optical trapping and manipulation of cells and biological structures and the subsequent biosensing in vivo will be a new trend in the following few years. However, the OFTs may induce mechanical damage when inserting the fiber into living samples. Therefore, the construction of biocompatible OFTs is of great importance, and maintains great application potential. Living biophotonic waveguide via the assembly of living bacteria or cells in vivo will provide new possibilities for the formation of biocompatible optical fibers [53,54]. Trapping, manipulation, sensing, and diagnostics in vivo will be possible with such optical fibers.

## Figures and Tables

**Table 1:** Comparison of single optical fiber tweezers (OFTs) with the conventional optical tweezers (COTs).

Items	Single OFTs	COTs
Key components	Laser source, tapered optical fiber	Laser sourcehigh NA objectivea number of optical components for beam expanding and steering
Fabrication and construction	Easy, simply fabricate a tapered optical fiber with different methods	Carefully design the beam path via the adjusting of beam expanding and steering components are necessary
Integration capability	Highly compact, can be integrated into microfluidic platform	Not compact
Manipulation flexibility	Highly flexible, trapped particles can be delivered to any designated positions by simply moving the fiber	Less flexible, trapped particles can only be manipulated by controlling the focus through beam steering and modulation elements incorporated with the high-NA objective
Suspension applicability	Wide, the fiber can be inserted into suspensions with any different directions and depths for trapping and manipulation	Suspension depth and direction is limited due to the focus generated by the high-NA objective.
